# A Paper on the Pathology and Treatment of Fever

**Published:** 1847-12

**Authors:** A. G. Henry

**Affiliations:** Pekin, Ill.


ILLINOIS AND INDIANA

MEDICAL AND SURGICAL JOURNAL.

Vol. II. NEW SERIES.—DECEMBER, 1847. No. 5.

### PART I.—ORIGINAL COMMUNICATIONS.

### ARTICLE I.

*A Paper on the Pathology and Treatment of Fever.* Read before the Peoria District Medical Society, at their Fall Session, 1847. By A. G. Henry, M. D., of Pekin, Ill., and ordered to be published in the Illinois and Indiana Medical and Surgical Journal.

The Pathology of Fever has been so often and so ably discussed, from the days of Hippocrates down to the present time, that nothing *new* can reasonably be expected from me on this occasion.

It may be said with truth, that almost every conceivable condition of the system has been referred to by learned Medical and Pathological writers, as the proximate cause of that morbid condition which we denominate Fever; and of the various systems which enter into the formation of the Animal Economy—each one in their turn have been referred to as being the first affected in the series of morbid derangements. The Profession generally, and especially gentlemen of this Society, are too well acquainted with the various theories that have been put forth to the world in relation to the Pathology of Fever, to render it necessary for me to allude to them in detail; consequently,

I shall occupy the time allowed me on this occasion, in setting forth, in as brief and comprehensive a manner as possible, the Pathology which I have adopted, upon which to base *a practice* which I claim to have been the first in the Medical World to introduce to the notice of the Profession. But while I claim to have *originated the practice,* I would not be understood as claiming any originality on the score of theory, for all the views which I shall advance at this time upon the Pathology of Fever, I am indebted for, to those bright and shining lights to whom the world is so much indebted for the amelioration of human suffering, and to none more than to the immortal Cullen and Sydenham.

It may be thought strange by some, but it is nevertheless true, that I am indebted to *my practice* and observation for anything like clear and satisfactory views of the Pathology of Fever, rather than to my *theories* for my practice. This may be regarded as unphilosophical, but to my mind, it is the only way by which real and substantial improvements can be made in the practice of the healing art. What is more in the way of the introduction of new curative agents in the treatment of disease, by the Profession, than is the peculiar theory which may have been imbibed by the practitioner, of the disease, and the modus operandi of the remedy proposed? If they condemn the practice, nothing short of occular demonstration can induce a trial of the remedy; for no individual verbal authority can stand against the generally received opinions of the Faculty; and it is well that it is so as a general thing. But there should be exceptions to this rule. When the remedy comes recommended by responsible medical men, who have formed their opinions of its efficacy from actual observation, and in the absence of all countervailing testimony, coming from equally responsible sources, it does seem to us that they should so far be received with favor, as to induce a fair trial of them, without their authors being required to accompany them with a theory more satisfactory than any that may have been previously promulged. If this is required of me in the present instance, I greatly fear gentlemen of this Society will never make a trial of my remedy. I am now satisfied, however, that I committed an error in presenting my new system of practice to the Profession, without some kind of a theory to support it, since the practice is in direct opposition to all the doctrines of the books, and contrary to the almost universal practice of the Profession.

I believe it may be said with truth, that all modern Pathologists agree that the Nervous System is the first to receive the impression of all those noxious influences that are supposed to act as remote or exciting causes in the production of fever. This, at least, is my doctrine. It is not my purpose to discuss the question of the relative importance of those insensible causes, which we call miasmatic influences, and the sensible causes, such as temperature, humidity, &c., in the production of the disease, but I content myself with the passing remark, that I attach far more importance to the latter than the former, for I am not disposed to look for *insensible* causes, when we have sensible ones enough to account for the phenomena.

If this, then, be true, it follows as a natural consequence, that the Nervous System is the first affected in the series of morbid derangements. This is most clearly proven in the first paroxysm of a common intermittent. The first thing complained of, is a general languor and debility dependent entirely upon functional derangement in this system. But let the paroxysms be repeated, and we have derangement in all the other systems. If we prevent a recurrence of the paroxysm by our quinine or other remedies, as a general thing, no sensible derangement is experienced in any other system—and my doctrine is, that quinine as a remedial agent in intermittents, acts directly upon the nervous centre. If it were not so, how shall we account for the fact, that it must be given until that peculiar effect is experienced in the head, on which it is conceded its periodic power is to be determined. If this effect is not produced, the paroxysm will return; and this fact is now becoming so well understood, that we regulate the quantity required in each individual case, by this effect—five grains producing it in one case, while twenty may be required in another; but all we give after this ringing in the ear is produced, is worse than thrown away.

The Intermittent form of Fever, is, in point of fact, the only pure and unmixed form we have; all others partake more or less of other forms of disease. Here we have Fever in its purity, and here we also have Dr. Cullen's "spasm of the extreme vessels," most clearly exemplified.

In the Typhus form, as well as in all others, we have fever and inflammation combined, and which is entitled to the precedence, never has, and probably never will be, satisfactorily determined. We may have fever, says Dr. Thompson, without inflammation, but we can never have inflammation without fever. In all forms of this mixed disease, if the case proves fatal during the irritative stage of the inflammatory part of it, no trace is left behind for the Pathologist; but if they are long protracted cases, actual lesion is found, sometimes in the brain or spine, or both; sometimes in the stomach and bowels, and at other times in some other minor viscera—and these facts have led to interminable disputes as to the seat of the disease—which all goes to prove most conclusively to my mind, that all that class of disease denominated fever, have no other local habitation but in the nervous system. That learned and justly celebrated Pathologist, Brousais, contends that all fevers are nothing more nor less than cases of gastro-enteritis; but Dr. Clutterbreck denounces this doctrine, and contends most learnedly that they are all cases of inflammation of the membranes of the brain. Other locations have been contended for with equal ability, and in the West and South, in latter days, the palm has been awarded to that class of Pathologists who locate the disease in the liver, and the result has been, that in this region, that greatest and best of all remedies, except opium, when properly used, has been degraded as a nostrum in the hands of the regular faculty, to a level with lobelia and number-six, and done more to encourage empiricism than everything else besides.

All these various theories, in my opinion, have been more the productions of fancy than fact. The pathology which locates the disease in the brain, comes nearer the true doctrine than any other, for the reason that it comes nearer the nervous system than any other. But it comes no nearer, in point of fact, to locating the disease originally in the nervous system, than does the doctrine of Brousais, the only difference being in nearness of proximity to the nervous centre; and in the stage of irritation large doses of opium can be given with as much safety as when the inflammation is in any other part; and the remedy is more loudly called for, because of the greater danger of a fatal termination, if the disease be not arrested at the onset. The notion which so generally obtains among medical men, that opium cannot be given with safety, when the head is the seat of irritation, is, in my opinion, entirely erroneous. It is only when the irritation has produced inflammation that opium becomes inadmissible, *and this rule is just as applicable to affections of other parts, as to the brain.* The symptoms of coma which usually show themselves in the latter stages of fever, as a general thing, are the results of nervous exhaustion. In such cases, the only remedy to be relied upon with any degree of confidence, is full doses of opium.

Let us for a moment inquire what are the first symptoms which usher in an attack of fever? Are they not such as imply derangement in the nervous system? and is it not true that they continue some time before any other symptoms occur that indicate derangement in any other system? Does not the experience of every medical man testify, that one local affection after another follows the progress of fever, showing conclusively that they depend, or rather result from a continuance of the primary irritations in the system first affected. No one will contend that derangement in the secretory system is the first in the series, neither is it found in the heart or the arteries. They are both secondary affections, evidently caused by the continuance of the primary irritation, wherever you may please to locate it.

If this be true, what then are the indications of cure? Should they be remedies that act most directly upon the secondary affections? or should they not be directed to the original affection?

What do we do when called to a patient laboring under disease produced by swallowing poison? Why we at once remove the irritating matter from the stomach. Instead of adopting this course, suppose we should allow the irritating cause to remain in the stomach, and set about trying to arrest the progress of the inflammation? What would be the result? Why, the inflammation would be increased more rapidly than we could subdue by the most vigorous application of our remedies.

Now I contend that the same principle applies to the treatment of fever. If we overlook the irritation in the nervous system, and direct our remedies to the secondary affections, as a general thing, every symptom becomes aggravated hour after hour, until our patient dies, or until we produce in the system, a new disease by our remedies, which the efforts of nature are competent to remove.

It is a well established law of the animal economy, that no two diseased actions can exist in the system at the same time, and it will not be denied that mercury cures fever by creating the specific action of the remedy; but when the febrile action is well established, it is very often impossible to substitute the action of our remedy, and we lose our patient. But if we do ultimately effect our object, it is at the expense of an immense amount of suffering, and a long, protracted confinement.

The Pathology which I contend for, and the Practice based upon it, saves to the patient this suffering, avoids the danger of a fatal termination, and subdues the disease nine times in ten, in the course of eight and forty hours.

But it is entirely unnecessary to indulge in theoretical speculations to prove that my views of Pathology are, in the main, correct, for the results of the Practice, about which there can be no mistake, most fully confirm them.

What is the great and leading indication in the treatment of fever? Is it not to subdue the preternatural heat on the surface, and has not a general perspiration ever been regarded as a highly favorable indication of cure? and can this be accomplished in any other way, than by the use of remedies that are calculated to allay the irritation upon which the "spasm of the extreme vessels" depend? And what class of remedies are resorted to to secure this desirable result? Are they not sedative? What is ice, but the most powerful sedative in nature, and does not the abstraction of blood produce a sedative effect, and are not these remedies generally resorted to for the purpose of subduing high arterial action, when no local afflictions forbid their use. Upon what system do sedatives act most directly? *Is it not on the nervous system?* If this be so, I insist that it is proof conclusive, that fever has its seat in that system. In the onset of fever we have no visible derangement in the Secretory System, and we sometimes have forms of fever, that continue for days and weeks without any evidence of derangement except in this system, and the heart and arteries, but they are certain to show themselves in time, unless the fever is subdued.

There is a great difference in fevers as relates to the extent of derangement in the nerves. In the Typhoid type, the disease is apparently confined to the nervous tissues. We have but little other functional derangement for many days, and the nervous symptoms predominate throughout the progress of the disease. This is no doubt attributable to the fact, that actual lesion takes place in some part of the spinal column early in the progress of the fever; while in other forms of fever, the irritation is located in some one or all of the other systems. We have forms of fever when the lungs are the seat of irritation; others when the stomach and bowels are most prominently affected—and we have a form of fever in which we have cerebral, thoracic, and abdominal affection, showing themselves simultaneously very soon after the first symptoms of nervous irritation are perceptible, and this form of fever will prove fatal nine times out of ten under the best plan of treatment recommended by the books. The Congestive forms of fever, now so common in this region, belong to this class of disease, and which I shall notice more fully before I am done.

In cases of this character, I suppose the impressions are made upon the nervous system in the same way that they are made in the less rapid and malignant forms, but owing to an unnatural degree of irritability in the nerves of the parts affected, we have a sudden rush of blood to the head, lungs, stomach, and bowels, or all together, which produces violent functional derangement in the organs, which speedily destroys life unless removed. In such cases, no trace of disease is left to guide the Pathologist, no perceptible lesion is found in any of the tissues, to account for the phenomena. That great derangement existed in the parts, no one can question, and that it is to be looked for in the nervous tissues is rendered highly probable from the following anatomical and physiological facts, that have been well established by Doctor Abercrombie in his researches into the diseases of the substance of the brain. He has shown that actual disorganization may take place without changing the appearance of the part materially. None of the appearances which are found in similar conditions of other tissues, are to be found in the nervous. In all the variety of inflammations we find ample traces of the disease to account for all the phenomena. Not so in fever; and yet the difference in the symptoms characterizing the two forms of disease, are more imaginary than real. In fever, we have general uneasiness, while in inflammation we have *pain* in particular parts, but in both we have increased heat and arterial excitement. Very soon, however, in fever, we have pain, and sometimes tumefaction in particular parts, and when this occurs, where are we to look for the distinction, except in the post-mortem appearances; and not even here in all cases, for where the patient lives long enough, we find actual lesion in the parts affected.

My doctrine is, that the difference consists mainly and solely in the fact, that in fever we have irritation and lesion in the nervous tissue, while in inflammations we have them in the other tissues. The morbid impressions in the one case are tangible to the senses, while in the other they are not.

The immortal Doctor Bell has demonstrated that the great bulk of the nervous tissue is destitute of sensation, consequently, in fever without local determinations, we have no pain, as we have in inflammations, but we do have what amounts to the same thing, a general uneasiness more distressing than actual pain. The nerves of nutrition and motion being destitute of sensation, the functions dependent upon them are affected imperceptibly, and hence the conclusion of some, that the diseased action originally exists in these functions. The nervous system being the most important of all others in the animal economy, is it not reasonable to conclude that it is more liable to disease than any other; and since all the functions depend upon it for their healthy action, is it strange that general disturbance should follow as a consequence of diseased action in the nervous system? a result we never fail to find in fever.

Having established (at least to my own satisfaction,) that fever is the result of disease in the nervous system, and that all other derangements are to be regarded as secondary affections, I shall proceed briefly to give my views of the practice which ought to be pursued in the commencement of the attack, and I know of no way by which I can make myself understood more clearly, than by reporting one or more cases (out of the hundreds I have treated on this plan, with the same uniform success,) of the different forms of fever found in this region of country, during the last ten years.

William S———, laborer, was attacked with Fever in February last. I saw him a few hours after he took to his bed at about 9 o'clock in the evening. He had been complaining of languor and debility during the day, but did not leave his work until about 4 in the evening, when he was suddenly seized with violent rigors, and so great was the muscular prostration, that he was unable to stand without help. He complained of general soreness over the whole surface. Pulse over 140, full, but easily compressed, with quick breathing, interrupted occasionally with a long heavy sigh; skin hot, but not dry. Complained of no pain except a slight pain in the head. Could fetch a full inspiration without causing pain. I opened a vein in the arm, more for the purpose of seeing the effect it would have upon the breathing, than from any expectation that it would relieve the symptoms; and after taking about 16 ounces, the symptoms were evidently aggravated. As usual in such cases, I gave him five grains of quinine, with five pills composed of one grain of opium and three of calomel, each, and left a powder composed of five grains of quinine with three of opium, to be given in six hours. I saw him two hours after, and found him sweating freely; pulse about 100, strong and full; breathing natural, and said he felt entirely well. Saw him the next morning at 8; had taken the powder as directed, and sweat freely during the night; pulse natural, pain and soreness gone; had not slept much during the night. I now gave him salts and senna, which operated freely in three or four hours, when I repeated the opium and quinine in doses of three grs. of each every six hours, until three doses had been taken. In the evening, found him sitting by the fire comparatively well, but complained of feeling extremely weak. Directed him to keep in doors for two days and live on soups, tea and toast, and at the end of that time he was at his work again as though nothing had happened. Some 10 other cases of this kind were treated in this way, except the bleeding, with the same results in the course of 8 or 10 days, during which time, 5 cases proved fatal under the evacuant plan of treatment in other hands in 48 hours, becoming entirely insensible after 20 or 24 hours from the commencement of the attack. The only patient I lost, was a child 5 years old. In this case, I gave a dose of Ipecac to relieve the difficult and rapid breathing. I returned in two or three hours after, and found my patient beyond the reach of remedy, and she died in 19 hours from the first symptom of indisposition, which was a slight chill, followed with fever and rapid breathing.

I regarded these cases as most violent and malignant forms of Typhus Fever, or, as they are sometimes called, cases of Typhoid Pneumonia, Cold Plague, Winter Fever, &c.,—differing only from the milder forms of Typhus, in the fact of being more rapid in their progress in consequence of the nervous system being as it were, *overwhelmed by the force of the exciting cause.* Hence the necessity or rather the advantage of combining the quinine with the opium and calomel. The quinine might have been omitted with safety, but in such cases I prefer using it. The calomel I know can be dispensed with without lessening the efficacy of the treatment, as I have demonstrated over and over again in the treatment of this form of fever, and I only give it as a matter of convenience, for its cathartic effect. The following case is in point. I would remark here, that I never resort to the quinine except in the malignant forms of the disease.

I was called to see Mrs. H———, who had recently lost in her family, a nephew and a daughter, after a lingering illness of what the doctor called the winter fever, both of which had been treated on the mercurial plan. She had been confined to her bed for five days, but had taken nothing but oil and pills of some kind. There was every symptom of Typhus Fever present, with slight affection of the lungs. She begged that I would not give her calomel, and not regarding it material, I promised I would not. I accordingly left her two powders of 4 grains of opium each, the second to be given eight hours after the first, and to drink hot sage tea freely until sweating was produced. Saw her the next day, much improved in appearance, and said she felt much better—had perspired freely all night. Directed a dose of oil and four grains of opium at bed time, with *three* grains eight hours after, with the same effect as before. Found her the next day greatly improved, and entirely free from fever. Repeated the oil, and left three grains of opium to be given at bed time. Did not see her again for three days. Found her sitting up free from fever, but very much troubled with a dry cough. Applied a blister to the chest, and left her the brown mixture. She recovered steadily but slowly, the cough not leaving her for ten or twelve days. Several other cases occurred in the same neighborhood, which I treated with opium in the same way, with the addition of the calomel, all of which were convalescent in forty-eight hours after the first visit, and no case was seen after the third visit.

Five cases, treated by other Physicians, proved fatal in the neighborhood that fall and winter (last winter). I am certain that I prescribed for two-thirds of all the cases that occurred, without losing a single case of fever in the vicinity.

I have now given a case of the mild, and most malignant forms of *Western* Typhus Fever. I now propose to give, for the purpose of more clearly illustrating my system of practice, one case of Congestive Intermittent, and one of common Remittent.

Some three weeks since I was called to see Mrs. S——. She had been taken the evening before with what she supposed to be a simple chill and fever. The chill had lasted some six or eight hours, accompanied, as she expressed it, "with a burning fever, and a feeling of freezing all the time," with the sensation of a loaded waggon running over her breast. She was lying in bed comparatively comfortable, and free from fever as she supposed, but expressed herself as alarmed for the consequence of the next chill, which she was expecting in the course of two hours. On feeling her pulse I readily detected that peculiar, but indescribable pulse, always found during the intermission in this form of disease. She said she felt tired, and when she attempted to sit up, she became sick at the stomach. I was satisfied from her general appearance that the next paroxysm would prove fatal, unless prevented or greatly mitigated, and there was no time to be lost. Accordingly, I administered in a little coffee, five grains of quinine with five of opium, and left five more of quinine with two of opium, to be given in six hours, directing her to drink hot tea freely until she got into a free perspiration, but before leaving the house she complained of feeling cold, and commenced breathing quick and laboriously. On going to the bed side, I found her hands cold; pulse small and rapid. The powder had only been swallowed some fifteen minutes, not long enough to produce any sensible effect upon the system, still I had every confidence that it would act in time to moderate the paroxysm. I applied a strong mustard poultice to the stomach, which acted promptly, when the breathing improved, but by this time the powder began to act. The pulse became slower and more distinct, drops of perspiration showed themselves upon the forehead, and in one hour from the time the dose was administered, she was perspiring freely, and said she felt better than she had for two days before; and being satisfied that she was safe, I retired for the night, repeating the direction to have the second powder given at the end of the six hours. The next morning I found her entirely comfortable—had not slept much during the night, but felt disposed to sleep then. I directed a dose of oil, which operated in two hours very freely, when I repeated the quinine in five grain doses without the opium, and left my patient convalescent. The next day she was up doing her work.

I am convinced that if the dose had been delayed one hour longer in this case, the paroxysm would have proved fatal, for I am satisfied from experience, that but little can be effected by way of mitigating the paroxysm after it is once fully formed, by internal remedies. If there is vigor enough in the vis-medicatrix natura to throw it off, you can save your patient by rightly improving the remission. I say *remission*, because I am satisfied that we have no *intermission*, such as we have in our ordinary intermittents, in this malignant form of fever, and I am equally satisfied that nothing but sedative doses of opium or quinine will save our patients—and of the two I prefer opium, for five grain doses of it prevents the paroxysm certainly, while the largest doses of quinine somtimes fail, particularly where the stomach is inclined to be irritable, which is often the case; and for the further reason, that the opium exerts no deleterious influence upon the system, which cannot be said with truth of 10 or 15 grain doses of quinine. From five to six grain doses of quinine, will, for all time to come, be my maximum dose. If I desire a sedative effect, I can procure it with more certainty with opium in four or six grain doses; *which I contend is the smallest dose that can be relied upon for the full sedative effect required for a case of fever.* The reason why so little benefit has been obtained from opium in the treatment of fever, heretofore, is to be attributed to the fact of its never having been given in full doses until given by myself.

When Dr. Cartwright, of Natchez, Mississippi, first proposed ten grain doses of quinine, the Medical World were as much shocked with its absurdity, as they now possibly can be at the dose of opium which I recommend; and it is but recently that it is conceded that it can be given in ten grain doses with comparative impunity, and to the extent of 40 and 50 grains in malignant forms of disease, without being destructive to life—and it is now universally admitted, that we can obtain two opposite effects by regulating the quantity. If we give it in one grain doses, repeated every hour, in a paroxysm of fever, we aggravate all the symptoms, and in all probability, before ten grains shall have been given, fatal congestion of the brain will have been produced. But if we were to give the ten grains at a dose, in precisely a similar case, none of these consequences will follow, but on the contrary we shall obtain full relief to all the symptoms by subduing the fever. This principle is more particularly applicable to opium than to quinine. Medical writers have long suspected that opium had an action different from that which had been usually attributed to it, and among them I would name the justly celebrated Pereira. [See his Materia Medica under the head of opium.] But no one, before myself, has had the temerity, as it has been called, to demonstrate the truth of the proposition by actual experiment upon the human system, in the treatment of a form of disease that occupies four-fifths of the time and talent of the Medical World; but when the practice shall take the place of the present abused mercurial system, (as it is bound, sooner or later, to do,) there will be a saving of thousands of lives, and an incalculable amount of human suffering.

But the great obstacle in the way of its adoption by the Profession, as I have said before, is the dread of fatal congestion of the brain, if given in such large doses. One says he has known fatal congestion of the brain, to follow the use of *one grain*, repeated once in two or three hours, until four or five grains had been taken; and they exclaim with uplifted hands and eyes: My God! if such results follow from one grain doses, what, but almost instant death, could be expected from a five grain dose. My friend, Dr. Golliday, in his learned "critique" upon a communication of mine, published in the Illinois and Indiana Medical Journal, some year since, on the treatment of fevers and inflammations without mercury, in which I urge the use of from 4 to 6 grains of opium, quotes various authorities to prove that my practice is not admissible in inflammatory affections, although it can be nowhere shown "in the writings of Dr. Christison, Dr. Sobernheim, Dr. Rankin, Dr. Williams, or Dr. Periera," that they had ever used a four or five grain dose of opium in their lives, in the treatment of the diseases alluded to, in which they condemn its use, when I claim to have given it hundreds of times in these doses with the happiest effects.

Now I would ask the Doctor how he can give the preference to their authority based on mere theory, without calling in question my veracity, a thing which I presume he had no intention of doing.

The Doctor does not seem to be aware of the fact, that I contend as strenuously as any other man, that anything short of a *full sedative dose*, (and by this I mean from four to six grains,) will as a general thing, do positive injury; and in that very communication which he very properly took the liberty of criticising, I laid down the proposition, that a two grain dose repeated once in two or three hours, was almost sure to do mischief, when a five grain dose given once or twice in twenty-four hours, would be productive of great benefit. As in the case with quinine, so with opium. Give it in one grain doses in a paroxysm of fever, every hour until four or five grains have been given, and the chances are two to one that you will greatly injure, if you do not destroy your patient. But give it in one dose, and none of these unpleasant consequences follow.

I have already extended my remarks far beyond anything I had intended in the outset. One word in relation to remittents, and I am done.

John R———, a German laborer, recently arrived in the country, was taken down the first of September with all the symptoms of a common bilious remittent. When I saw him he had been in bed 24 hours with high fever, hot, dry skin, great thirst, and violent pain in the head and back. I found him the second day about noon, with all these symptoms aggravated; pulse 120, full and strong, tongue dry and glossy, and very red at the point. Bowels had not been moved for thirty-six hours. I gave him an emetic of Ipecac in broken doses, at long intervals, say half an hour, for the purpose of securing a full emetico-cathartic effect. Saw him four hours after and found that his stomach and bowels had been thoroughly evacuated, but with little relief to the symptoms. I now gave him five pills composed of opii i grain, calomel iii grains each; directed him to drink freely of hot tea, and left him for the night with every confidence of finding him in the morning entirely relieved. I found him as anticipated, free from fever, the pain in the head and back entirely gone, tongue moist and heavily coated with a loose, white fur. Said he got easy about two hours after taking the pills, and had sweat freely all night, and expressed himself as feeling almost well. Left him three five grain doses of quinine, to be taken once in four hours during the day, and to drink as much water gruel as he pleased. Found him in the evening down at the table eating his supper; said he was very hungry, and felt entirely well, "except a little weak." His bowels had been freely moved during the day by the calomel. On the 4th of September he was again at his work as usual.

I have selected this case from among many others treated in the same way, for the reason that he complained more of his head and back than any other case I have treated this season, by way of showing Dr. Golliday and others, that a five grain dose of opium can be given, not only with safety, but with decided advantage, even when there was "strong symptoms of congestion of blood," and that too, to the brain and spinal column.

Now, supposing this case had been treated upon the usual plan; bleed first, give the emetic and the calomel at night without opium, does any gentleman suppose that he would have passed a comfortable night, been entirely free from fever in the morning, and at his work the next day? Would it not have been more likely that he would have spent a distressing night, with a temporary intermission in the morning, to be followed with an exacerbation in the evening, and this repeated day after day until mercurial action was established, or he beyond the reach of all remedy. Such has been the result of similar cases treated on the mercurial plan, at the *same time, and in the same neighborhood*, and such would have been the result had he fallen under my treatment ten years ago.

Do not such results go to prove beyond all question, that large doses of opium possess a power in the treatment of fever, greater than any other known remedy. The remedy which I once regarded as my sheet anchor, I can now dispense with entirely without material detriment to the treatment. But I do not propose to throw it aside; on the contrary, I still regard it as one of the best and most convenient *purgatives* in the whole materials of medicine. I only ask that it shall no longer be relied upon as the main remedy in the treatment of fever; for the reason that we have, in large doses of opium, one better adapted to meet the indications of cure.

In conclusion let me caution those of my professional brethren who would give the remedy a trial, not to delay a resort to it *until they have exhausted every other known remedy*, and when the case is beyond the reach of human agency. If you use it at all, use it in the early stage as I have directed, and my word for it you will not find cause to regret it.

